# The Father of English Surgery

**Published:** 1898-02

**Authors:** 


### THE FATHER OF ENGLISH SURGERY.

John Ardene, a contemporary of Chaucer, Wycliffe, Froissart, Petrarch and Guy de Chauliac, belongs to a period of extreme interest in the evolution of general and surgical literature, and it will be seen that he within his sphere gave no mean aid toward the advance of a higher culture. Of biographic facts we have but little to record, but it is known that he was born in 1307 and practiced at Newark in Nottingham from 1349, the year of the plague, until 1370, when, at the age of sixty-three years, he settled in London. Thenceforth he appears to have devoted his days to the publication of his experiences, in the form of treatises upon medicine and surgery. It is surmised that he had been previously attached for a time to the English forces during the French wars in the capacity of field surgeon, for there is no doubt that he was well acquainted with France and its language, and that he had an extensive experience in the treatment of wounds; but although he calls himself *chirurgus inter medicos* there is nothing to show that he had possessed a Master's degree or any formal license for the exercise of his calling. However this may be, his writings prove that he was a man of clerkly attainments, with a good knowledge of Latin and French, and well read in the available literature of his profession, quoting freely from the works of the medieval surgeons, the Arabs, and even from the Greeks. That he achieved fame as a surgeon is no less certain. He refers with pardonable pride to a number of distinguished patients whom his ministrations had restored to health, and he is said to have received from the Black Prince a grant of land in Connaught, and with it the right to prefix the noble particle "de" to his name. It is hard to say whether the position of an Irish landlord was more enviable then than now, but the colossal fees he was able to command from his wealthy clients probably rendered him independent of his Hibernian rentals. In this case, as in that of Guy de Chauliac, the literary labors which have preserved his memory were the outcome and not the cause of his success, for it was not until he had won an honorable repose that he found leisure to give the world the secrets of his practice. His writings, couched in fair Latin and dealing with nearly the whole of medicine and surgery as they were then known, were noteworthy productions of a reactionary but still illiterate age, and they appear to have been duly valued during the next three centuries, although none received the honors of the press except an abstract translation of an essay on fistula, which was printed in 1588, by John Read, as an appendix to a translation of Franciscus Arceus on wounds.

When Ardene was in his prime there was but one scientific center in Europe where any pretense of surgical teaching was maintained. The once famous school of Salerno had already become effete. The Italian revival under William of Saliceto, Theodoric, Lanfrank, Modini and others, had given place to the old stagnation against which Bologna had made a valiant fight before sinking into premature decay. Paris, too, after listening to the teaching of Lanfrank and of Henri de Mondeville, had absolutely turned her face to the wall, and Montpellier alone, thanks in part to its geographic position, which brought it within nearer touch of Arab learning, was liberal enough to sanction the cultivation of anatomic and surgical science, not, indeed, within the walls of its university, but in extramural classes, in which, among others, Guy de Chauliac, the greatest surgical figure of the age, took a leading part. Even this tolerance soon died out, and Montpellier went the way of Salerno, Bologna and Paris, but not until the work of Guy de Chauliac and John Ardene was ended. In England the age of Chaucer and Wycliffe made no provision whatever for medical progress, and there was no school of anatomy or surgery throughout the land, and no English contributors to the literature of the subjects could be said to exist, if we expect such theoretic references as may be found in the works of Gilbertus Anglicanus and John of Gaddesden, written, like all medical treatises of the time, in Latin. It is true that the writings of the Arab physicians, which gave an imperfect sketch of Greek surgery, and those of William of Saliceto, Roger, Theodoric, the Four Masters, Lanfrank, de Mondeville, and, after 1363, the masterpiece of compilation, the Chirurgia Magna of Guy de Chauliac, were also procurable in Latin guise, but the knowledge to profit by them was rare indeed in the ranks from which the representatives of English surgery were drawn. In the fourteenth century the lot of those of our countrymen who fell in need of our surgical ministrations was a sadly precarious one. The art was regarded by the educated physicians then, and for centuries later, as beneath their dignity. As expressed by our Elizabethan surgeon, John Read, in the doggerel which he and his fellows loved to prefix and append to their professional writings:

"Chirurgery moreover is
Abhorred of the Phisition,
Who doth esteeme it as a thing
Too vile for his profession."

His writings convey a vivid picture of the man. It is easy to see him as the leech of courtly and obliging manners, sober in attire and moderate in speech, and endowed with a self-restraint born of self-respect, with a tact that bore him well through the many difficulties that must have beset the thorny path of the *chirurgeon* in his day, and with a strong common sense that never rose too near the dangerous level of genius; a goodly man and true, kindly and honest, but shrewd withal, with a quick eye to the main chance and a capacity of raising expediency to the dignity of principle. He was through all a surgeon, a scholar and a gentleman, and in the records he bequeathed he stands before us as one whom we, his professional descendants, may accept with pride and veneration as the Father of English Surgery.—*London Lancet.*

